# Improved and synchronized maturation of Norway spruce (*Picea abies* (L.) H.Karst.) somatic embryos in temporary immersion bioreactors

**DOI:** 10.1007/s11627-018-9911-4

**Published:** 2018-07-20

**Authors:** Nazmul H. A. Mamun, Cyrus K. Aidun, Ulrika Egertsdotter

**Affiliations:** 1 G. W. Woodruff School of Mechanical Engineering, Georgia Institute of Technology, 801 Ferst Drive, Atlanta, GA 30332 USA; 2 Parker H. Petit Institute for Bioengineering and Bioscience, Georgia Institute of Technology, 315 Ferst Drive NW, Atlanta, GA 30332 USA; 3 Department of Forest Genetics and Plant Physiology, Umeå Plant Science Center, Swedish University of Agricultural Sciences, 901 83 Umeå, Sweden

**Keywords:** Dispersion, Synchronization, Yield, Somatic embryogenesis, Norway spruce *Picea abies* (L.) H.Karst

## Abstract

Somatic embryogenesis offers many benefits for clonal propagation in large-scale plant production of conifers. A key rate-limiting step is the conversion from early-stage somatic embryos in pro-embryogenic masses (PEMs) to the maturation stage. Immature embryos in PEMs are present at different developmental stages, where some are unable to respond to the maturation treatment, thus limiting yields of mature embryos. Synchronization of early somatic embryo development in PEMs could greatly improve subsequent yields of mature embryos. A temporary immersion bioreactor designed for Norway spruce (*Picea abies* (L.) H.Karst.) was used in this study. Through a specific system for dispersion, connected tissue of PEMs, composed of immature embryos grown in liquid medium in the temporary immersion bioreactors or on solid medium as a control, was dispersed and redistributed in a more uniform spatial arrangement. It was demonstrated that development of mature embryos could be significantly stimulated by dispersion, compared to controls, in both medium types. Synchronization of maturation was evaluated by a statistical approach. The present study shows that the yield of mature embryos from dispersed PEMs was three to five times higher than that from non-dispersed controls in three of four cell lines of Norway spruce tested, both in bioreactors and on solid medium.

**Electronic supplementary material:**

The online version of this article (10.1007/s11627-018-9911-4) contains supplementary material, which is available to authorized users.

## Introduction

Somatic embryogenesis (SE) offers many advantages as a clonal propagation method for large-scale, commercial production of conifer plants for forestry operations (Nehra *et al.* 2005). The cost of production has, however, long been hampering full implementation, particularly in high labor cost locations like Europe (Lelu-Walter *et al.* 2013). Recent efforts to automate the most cumbersome steps of the SE process provide more cost-effective plant production methods, requiring less manual labor, and will further aid in expanding such commercial operations to high labor cost regions (Aidun and Egertsdotter 2012). In addition to automating the SE methods, improved yields from key steps of the SE developmental path, *e*.*g*., maturation and germination (Dobrowolska *et al.* 2017) would greatly reduce the cost of production.

A key rate-limiting step of the SE developmental process concerns maturation and the yield of mature embryos. It is well known that somatic embryo cultures are not synchronized during development. Increasing the degree of synchronization during maturation of somatic embryos would significantly increase the yield of plants and reduce the losses at an early stage of plant development.

One approach to increasing the degree of synchronization of the maturation process is to divide the culture of immature, proliferating embryos into smaller aggregates, before the maturation treatment starts. Typically, immature somatic embryo cultures are composed of cell aggregates of different sizes. Several studies have shown a positive effect on synchronized development from size fractionation of early-stage somatic embryo cultures in angiosperms, *e.g.*, in *Daucus carota* L. (carrot; Fujimura and Komamine 1979), *Citrus sinensis* (L.) Osbeck (orange; Souza *et al.* 2011), and *Fraxinus angustifolia* Vahl (narrow-leafed ash; Tonon *et al.* 2001).

In conifers, the corresponding stage of early somatic embryo development differs from that in angiosperms by the callus-like tissue (so-called pro-embryogenic masses (PEMs)) composed of collections of early-stage somatic embryos (Filonova *et al.* 2000). Larger, connected aggregates of PEMs are formed by individual early-stage embryos through growth and repetitive development of new early-stage somatic embryos.

Multiplication rates of PEMs are highest in liquid suspension cultures (von Arnold *et al.* 2002). It is assumed that agitation of the liquid medium breaks up this connected tissue and releases individual embryos and smaller aggregates of PEMs that can then continue to proliferate without hindrance from adjacent tissue (Egertsdotter 1998). However, most conifer SE cultures are matured on solid medium, as maturation frequently fails under liquid suspension culture conditions.

In the present study, a glass disperser tube supported by a peristaltic pump was used to disperse the clusters of PEMs to obtain smaller aggregates of PEMs (Aidun and Egertsdotter 2011). Such a disperser as part of the SE fluidics system has been demonstrated to provide effective dispersion of embryogenic tissue without damaging effects (Aidun and Egertsdotter 2012).

Temporary immersion bioreactors offer a flexible culture system, by combining the benefits of PEM-multiplication in liquid medium with the solid support required for maturation, as demonstrated for *Coffea arabica* L. (coffee; Etienne and Berthouly 2002), *Theobroma cacao* L. (cacao tree; Niemenak *et al.* 2008), and *Elaeis guineensis* Jacq. (oil palm; Gomez *et al.* 2016). In conifers, successful use of temporary immersion bioreactors has only been reported for *Abies nordmanniana* (Steven) Spach (Businge *et al.* 2017) and Norway spruce (Aidun and Egertsdotter 2012).

The aim of the present work was to study the effects on maturation yield and synchronization in Norway spruce cultures, when a partially automated disperser system, suitable for large-scale applications, was used in combination with a novel temporary immersion bioreactor for multiplication of PEMs and production of mature embryos. Synchronization was measured by mature embryo size distribution and was evaluated by different statistical approaches. Non-dispersed cultures and cultures on solidified culture medium were used as controls. The hypotheses that (a) dispersion of PEMs before maturation treatment and/or (b) proliferation and maturation in the temporary immersion bioreactor designed for conifer SE would increase the yield and synchronization of mature embryos were tested.

## Materials and methods

### Plant material

Four embryogenic cell lines, 11:12:02, 11:12:04, 09:73:06, and 09:77:03 of Norway spruce (*Picea abies* (L.) H.Karst.) initiated and established by standard procedures (von Arnold and Clapham 2008) were used in this study. Briefly, the cell lines were maintained through proliferation/multiplication of PEMs in the dark, by subculture every 2 wk on solid, half-strength LP (½ LP) medium (von Arnold and Eriksson 1981), supplemented with 2.21 mg L^−1^ 2,4-dichlorophenoxyacetic acid (2,4-D), 1 mg L^−1^ N6-benzyladenine (BA), and 10 g L^−1^ sucrose in 9-cm-diameter Petri plates. For maturation, PEMs were transferred to pre-maturation medium (DKM; Krogstrup 1986, without growth regulators) for 7 d before transfer to DKM maturation medium supplemented with 16 mg L^−1^ abscisic acid (ABA and 30 mg L^−1^ sucrose (Dobrowolska *et al.* 2017)). Proliferation and maturation media were adjusted by HCl and NaOH to pH 5.8 before autoclaving at 121 °C and 103.4 kPa. Maturation lasted 8 wk, during which the cultures were subcultured biweekly to fresh DKM maturation medium. Liquid media of the same composition, but without gelling agent, were autoclaved and used for the bioreactor cultures described in the ‘Experimental set up’ section.

Harvested mature somatic embryos from the experiments were desiccated for 3 wk in darkness and high humidity (100%) in a closed Petri plate together with a smaller Petri plate with water according to standard procedures for desiccation of somatic embryos of Norway spruce (von Arnold and Clapham 2008). Desiccated mature embryos were placed on solidified germination medium for 1 wk in darkness, 2 wk in continuous red light (wavelength 660 nm; TL-D 18W/15, Philips, Stockholm, Sweden) at 5 μmol m^−2^ s^−1^ at 20 °C, and then moved under continuous white fluorescent tubes (Fluora L 18W/77, Osram, Johanneshov, Sweden) at 100–150 μmol m^−2^ s^−1^ (Dobrowolska *et al.* 2017). The germination medium had the following composition per liter: 764 mg KNO_3_, 173 mg NH_4_NO_3_, 381 mg KH_2_PO_4_, 533 mg MgSO_4_^**.**^7H_2_O, 83 mg CaCl_2_·2H_2_O, 30 g sucrose, 0.5 g casein hydrolysate, 1 mg thiamine, 50 mg inositol, and for solidified medium, 3.5 g gelrite™. Iron and microelements were the same as for DKM medium. The pH was adjusted to 5.8 prior to autoclaving. All steps of the culturing process, from proliferation to germination, took place in culture rooms set at 22 ± 3°C. All chemicals were obtained from Sigma-Aldrich Sweden AB, Stockholm, Sweden.

### Experimental set up

Effects of dispersion on PEM proliferation and embryo maturation were studied in cell lines 11:12:02, 11:12:04, 09:73:06, and 09:77:03 on solid medium and in cell lines 11:12:02 and 11:12:04 in temporary immersion bioreactors. For culture on solid medium, 0.5 g of PEMs was dispersed in 40 mL ½ LP liquid medium of the same composition as used for proliferation, using the Mamun (2015) disperser system at a flow rate of 2500 mL min^−1^ under sterile conditions. Three replicates each of cell lines 11:12:02, 11:12:04, 09:73:06, and 09:77:03 were set up by spreading 40 mL of the dispersed PEMs through pipetting onto a filter paper (Whatman Grade 2 Qualitative Filter Paper Standard Grade, circle, 70 mm, GE Healthcare—Whatman, VWR, Umeå, Sweden) placed on solid proliferation medium in a 9-cm Petri plate. Excess medium fluid was removed by pipetting. As a control, the same amount of non-dispersed aggregates of 3- to 5-mm-diameter tissue clumps of PEMs were transferred by forceps to solid proliferation medium in Petri plates. In total, for the experiments on solid medium, each cell line was represented by three Petri plates with dispersed cultures and three Petri plates with non-dispersed cultures.

For bioreactor culture, PEM aggregates were dispersed using the same dispersion system as used for solid cultures. Six bioreactors of cell lines 11:12:02 and 11:12:04 were set up by dispersion of 2 g of PEM aggregates into 50 mL ½ LP liquid medium spread onto the bioreactor screens. As a control, the same amount of non-dispersed aggregates (3–5 mm in diameter) of PEMs were transferred by forceps from solidified proliferation medium in Petri plates to each of six replicate bioreactors for either cell line. In total, for the experiments in bioreactors, each cell line was represented by six bioreactors with dispersed cultures and six bioreactors with non-dispersed cultures.

Temporary immersion bioreactors (Mamun 2015; Businge *et al.* 2017) were used for production of mature somatic embryos through a first step of proliferation of embryogenic masses (both dispersed and non-dispersed), followed by maturation in the same bioreactor. Immersion periods were set to a period of 1 min in every 12 h for all developmental stages. After 12–14 d, the proliferation medium was replaced by pre-maturation medium for 7 d, followed by maturation medium for 8 wk. During maturation, the bioreactors were subcultured biweekly, by replacing the maturation medium in the flasks associated with the bioreactor with fresh culture medium.

### Methods for evaluation of embryo maturation and synchronization

**Figure 1.**
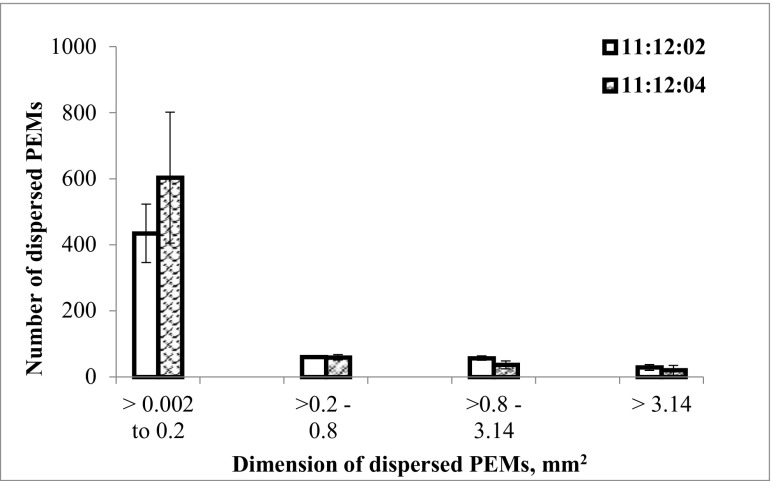
Size distribution of dispersed aggregates of pro-embryogenic masses (*PEMs*) of two cell lines of Norway spruce (*Picea abies*). The clusters of PEMs of cell lines 11:12:02 and 11:12:04 in liquid medium passed through the dispersion system several times (until all the dispersed pieces could pass the disperser without blocking) and then digital images were collected. The images were analyzed using ImageJ software to estimate the size distribution of dispersed PEMs. The *error bars* show standard deviations for three replicates of each cell line.

To study the effect of dispersion on embryo maturation, PEMs were multiplied in proliferation medium for one subculture cycle (14 d), and then taken through the pre-maturation and maturation steps, as described above. After 8 wk of maturation, samples of tissue composed of PEMs and mature embryos were randomly collected from each bioreactor and transferred to Petri plates. Three samples were collected from each dispersed bioreactor, as were several tissue clumps from non-dispersed bioreactors. All embryos were then harvested from the collected tissue samples under a microscope (Nikon SMZ-10, Nikon Corp., Japan) using ×10 magnification. The total number of mature embryos in each replicate and the number of mature embryos per gram of fresh weight (gFW^-1^) at the start of the proliferation treatment were determined. The proportion of embryos collected out of the total number of embryos present in the bioreactor was estimated, based on the area of tissue sampled compared to the total area covered by tissue in the bioreactor. The sampled area was measured by image analysis of an overview photo of the bioreactor, after the samples had been removed. The length of each harvested embryo was measured using image analysis and analyzed as described in (Fig. 1).

### Image analysis of PEMs, bioreactor cultures, and mature embryos

Images of dispersed PEMs, cultures in bioreactors at the end of maturation, and mature embryos after harvest were acquired by a digital single-lens reflex (DSLR) camera (Nikon D5100, Nikon Corp., Tokyo, Japan). At the start of the experiments, three samples of dispersed PEMs submerged in liquid medium were collected from each bioreactor, poured into Petri plates, and photographed for estimating the size distributions. Each sample contained 651 ± 155 dispersed PEMs. The area occupied by each dispersed PEMs was obtained from image analysis using ImageJ 1.48v (National Institute of Health, Bethesda, MD) and was considered as the dimension of a dispersed PEMs. Results from the replicates were combined to get the estimated mean and standard deviation of the size of dispersed aggregates in each cell line (Fig. 1).

At the end of maturation, bioreactors were opened, and two images of each bioreactor culture were analyzed in ImageJ to estimate the area occupied by the harvested mature embryos. Since the embryos had developed rather homogeneously from the dispersed PEMs, the number of embryos gFW^−1^ of PEMs in dispersed bioreactor was estimated from the number of harvested embryos and the area occupied by them.

A total of 1037 and 1176 images of mature embryos were captured from dispersed and non-dispersed PEMs, respectively, of cell line 11:12:02, and 727 and 573 images of mature embryos from dispersed and non-dispersed PEMs, respectively, of cell line 11:12:04. On solid culture medium, images of somatic embryos from 24 plates of dispersed and non-dispersed cultures, containing an average of 129 and 78 mature embryos per plate, respectively, were analyzed. The images were trimmed using GIMP 2.8.10 (GNU Image Manipulation Program, a free and open-source graphics editor, https://www.gimp.org/) and converted into a binary image using ImageJ. For each embryo, the length of the major axis of the ellipse drawn around the embryo was acquired in ImageJ. The axis length was considered as the length of an embryo. The number of embryos was then obtained for each of the embryo length intervals of 0.0–1.0, 1.0–2.0, 2.0–3.0, 3.0–4.0, and 4.0–5.0 mm, giving δ as 1 mm.

### Statistical analysis

Differences between dispersed and control cultures were measured as the: (a) number of mature embryos gFW^−1^ of PEMs; (b) coefficient of variation (CV) for number of mature embryos per bioreactor; (c) average length of embryos, (*q̄*), in a length interval; (d) width of the normalized length distribution function, *φ*(*q̄*), at 50, 66, and 75% of maximum; (e) CV of lengths of embryos; and (f) confidence interval of CV. The number and length of embryos, and the width of the curve, *φ*(*q̄*), were normally distributed (see Supplementary document published online for the details and *φ*(*q̄*) plots).

## Results

**Figure 2.**
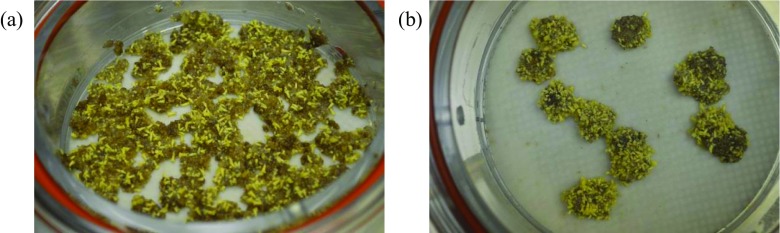
Pro-embryogenic masses (PEMs) with mature Norway spruce (*Picea abies*) embryos of cell line 11:12:04 after 8 wk on maturation medium in bioreactors (*a*) from PEMs dispersed at the start of the bioreactor culture and *(b*) from non-dispersed PEMs.

After dispersion of PEMs, proliferation appeared to increase relative non-dispersed tissues in all tested cell lines, both on solid medium and in bioreactors (Fig. 2). The initial average weights of dispersed and non-dispersed PEMs in bioreactors were 2.15 and 2.19 g, respectively. The proliferating cultures were not weighed during the 2 wk of proliferation in the experiments, as this would have caused too much disturbance to the cultures during removal from the bioreactors for weighing, and the remaining liquid medium inside the bioreactor would have further added to the measured weight. Instead, the average weights at the end of maturation treatment were measured for dispersed PEMs in 11 bioreactors, as 5.54 × (cell line 11:12:02) and 9.25 × (cell line 11:12:04), and of non-dispersed PEMs in 11 bioreactors, as 2.05 × (cell line 11:12:02) and 2.14 × (cell line 11:12:04), of initial weights. Two bioreactors of cell line 11:12:04, one each of dispersed and non-dispersed, were lost to contamination.

### Effect of dispersion on yield and synchronized development of mature embryos

**Figure 3.**
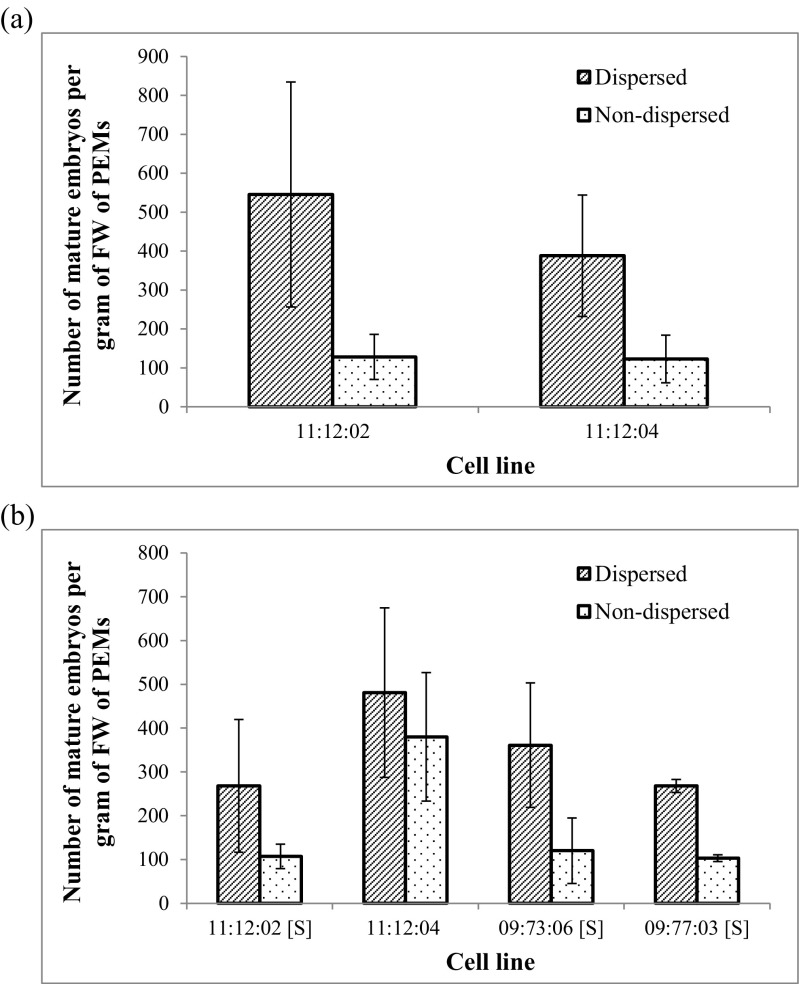
Yield of mature embryos. *Bar graphs* show the number of matured embryos developed per unit gram of starting fresh weight (*FW*) from *dispersed* and *non-dispersed* pro-embryogenic masses (PEMs) of various Norway spruce (*Picea abies*) cell lines. (*a*) Mature embryo yields in cell lines *11:12:02* and *11:12:04* cultured in bioreactors. The differences between *dispersed* and *non-dispersed* were statistically significant in independent two-sample *t* test with *p* value < 0.01. (*b*) Number of mature embryos per gram *FW* of cell lines *11:12:02*, *11:12:04*, *09:73:06*, and *09:77:03* on solid medium. The *error bars* show the standard deviation for three replicates. Number of mature somatic embryos developed from 1 g of fresh *dispersed* and *non-dispersed PEMs* are statistically different by independent two-sample *t* test at *p =* 0.10 for cell lines followed by *[S]* in abscissa.

The yield of mature embryos in bioreactors was higher from dispersed PEMs than from non-dispersed PEMs in both cell lines (Fig. 3*a*). The average number of mature embryos gFW^−1^ of dispersed PEMs was 4.25 times higher in cell line 11:12:02 and 3.15 times higher in cell line 11:12:04. Dispersion also resulted in a significant increase in yield of mature somatic embryos on solid medium for cell lines 11:12:02, 09:77:03, and 09:73:06 (Fig. 3*b*). The CVs of yield of mature embryos did not vary significantly between bioreactor replicates and that of plate cultures. For cell line 11:12:02, the CV of yield of mature embryos in bioreactors was 0.53 (dispersed) and 0.45 (non-dispersed), and in plate cultures was 0.57 (dispersed) and 0.26 (non-dispersed). For cell line 11:12:04, the yield CV values for bioreactors were 0.4 (dispersed) and 0.5 (non-dispersed), and for plate cultures, these values were 0.4 (dispersed) and 0.39 (non-dispersed).

Due to the large number of mature embryos present, on average 15% in 11:12:02 and 18% in 11:12:04 of mature embryos were harvested from each bioreactor of dispersed PEMs. From non-dispersed bioreactors, on average 75% in 11:12:02 and 59% in 11:12:04 of the mature embryos were harvested from each bioreactor. The lengths of the mature embryos were measured, and the length-data statistically tested by width of the curve of φ(q̅) at 50, 66, and 75% of maximum, CV of lengths of embryos, and confidence interval of CV.

The average embryo was significantly longer in dispersed than in non-dispersed PEMs in bioreactor-cultured cell line 11:12:02, but not in cell line 11:12:04 (Table 1). In solid culture, average lengths of mature embryos from dispersed and non-dispersed PEMs of each cell line did not vary significantly within cell lines (Table 1).

**Table 1.** Lengths of mature somatic embryos of Norway spruce (*Picea abies*) developed from dispersed and non-dispersed pro-embryogenic masses (PEMs) of different cell lines cultured in bioreactors (*BR*) or on solid medium (*SM*)

Cell line	Culture condition	Length of embryos (mm) (Mean ± SD)		Coefficient of variation	
		BR	SM	BR	SM
11:12:02	Dispersed	3.05 ± 0.74	2.06 ± 0.42	0.24	0.20
	Non-dispersed	2.92 ± 0.77	2.47 ± 0.67	0.27	0.27
11:12:04	Dispersed	2.77 ± 0.87	2.55 ± 0.63	0.31	0.25
	Non-dispersed	2.73 ± 0.84	2.32 ± 0.50	0.31	0.22
09:73:06	Dispersed	–	2.94 ± 1.04	–	0.35
	Non-dispersed	–	3.54 ± 1.29	–	0.36
09:77:03	Dispersed	–	2.42 ± 0.72	–	0.30
	Non-dispersed	–	2.12 ± 0.50	–	0.24

The difference is statistically significant for bioreactors by independent two-sample *t* test for cell line 11:12:04 (*p* < 0.01), but not for 11:12:02 (*p* = 0.49) or for solid cultures

The degree of synchronization was estimated by comparing the difference between full width at half maximum (FWHM) of six (five for 11:12:04) bioreactors containing dispersed PEMs and six (five for 11:12:04) bioreactors culturing non-dispersed PEMs (Table 2, Fig. 2). By performing independent two-sample *t* tests, the results suggested that there was a statistically significant (*p* = 0.05) difference in maturation yield from dispersed and non-dispersed PEMs in bioreactors. Similarly, the difference in widths of $\varphi \left(\overline{q}\right)$ at 66% (*p =* 0.05) and 75% (*p* = 0.07) of maximum were also statistically significant. At 66% of maximum of the normalized distribution function, the widths were 1.08 and 1.25 mm for dispersed and non-dispersed PEMs, respectively, and at 75% of maximum of φ(q̅), the corresponding widths were 0.88 and 1.03 mm. The shorter widths of the function φ(q̅) at three different locations in dispersed PEMs of cell line 11:12:02 represented the synchronized development of somatic embryos due to dispersion. However, in cell line 11:12:04, full width values at 50% (*p* = 0.16), 66% (*p* = 0.27), and 75% (*p* = 0.34) of maximum of the function $\varphi \left(\overline{q}\right)$ of dispersed PEMs were larger than those values for non-dispersed PEMs (Table 2). Hence, dispersion did not improve the level of synchronization of somatic embryo development from dispersed PEMs of cell line 11:12:04.

**Table 2:** Evaluation of synchronization by comparisons of widths of the plots of normalized distribution function, $\varphi \left(\overline{q}\right),$ for mature Norway spruce (*Picea abies*) somatic embryo lengths. The table shows average values and standard deviations of widths at 50, 66, and 75% of maximum of the normalized distribution function, $\varphi \left(\overline{q}\right),$ for lengths of mature embryos from dispersed and non-dispersed PEMs of different cell lines in bioreactors (*BR*) and on solid medium (*SM*)

Cell line	Culture condition	FWHM (50% of maximum)		Width $\varphi \left(\overline{q}\right)$ at 66% of maximum		Width $\varphi \left(\overline{q}\right)$ at 75% of maximum	
		BR	SM	BR	SM	BR	SM
11:12:02	Dispersed	1.47 ± 0.15	0.97 ± 0.14	1.08 ± 0.11	0.71 ± 0.10	0.88 ± 0.086	0.56 ± 0.07
	Non-dispersed	1.65 ± 0.2	1.45 ± 0.46	1.26 ± 0.19	1.02 ± 0.33	1.025 ± 0.19	0.85 ± 0.3
11:12:04	Dispersed	2.0 ± 0.28	1.49 ± 0.22	1.54 ± 0.22	1.24 ± 0.21	1.26 ± 0.19	1.06 ± 0.21
	Non-dispersed	1.81 ± 0.28	1.09 ± 0.12	1.434 ± 0.3	0.85 ± 0.08	1.19 ± 0.3	0.70 ± 0.07
09:73:06	Dispersed	–	1.91 ± 0.14	–	1.39 ± 0.19	–	1.11 ± 0.18
	Non-dispersed	–	2.47 ± 0.57	–	1.88 ± 0.45	–	1.56 ± 0.41
09:77:03	Dispersed	–	1.45 ± 0.2	–	1.16 ± 0.2	–	0.96 ± 0.18
	Non-dispersed	–	1.21 ± 0.18	–	0.96 ± 0.13	–	0.82 ± 0.1

*FWHM* full width at half maximum

Using the same method to evaluate synchronization, dispersion was also found to increase the level of synchronization in cell lines 11:12:02 and 09:73:06 on solid medium (Table 2, Fig. 3). The means of FWHM of φ(q̅) of cell line 09:73:06 were 1.91 and 2.47 mm for dispersed and non-dispersed, respectively, with δ = 1 mm. For the cell line 11:12:02, the averages of FWHM of φ(q̅) of three replicates containing dispersed PEMs and another three replicates culturing non-dispersed PEMs were 0.97 and 1.45 mm, respectively, with δ = 0.5 mm for better resolution of the distribution function. Full widths of φ(q̅) at 66 and 75% of maximum were also verified for both dispersed and control cultures (Table 2), which further suggested similar levels of synchronization of mature embryos after dispersion in cell lines 11:12:02 and 09:73:06. Mean values of the full width at 50, 66, and 75% of maximum of the function $\varphi \left(\overline{q}\right)$ for dispersed aggregates of PEMs were larger than those of non-dispersed aggregates of cell lines 11:12:04 and 09:77:03 (Table 2). Therefore, it would appear that dispersion did not improve the level of synchronization of mature somatic embryo development from dispersed PEMs of cell lines 11:12:04 and 09:77:03 on solid culture medium.

The coefficient of variation could be considered as another measure of synchronization of embryo development, since it determines whether the data (here the length of embryos) were more spread out from the mean. In bioreactor cultures of cell line 11:12:02, the CV in case of dispersed PEMs was smaller (0.24 with 90% confidence interval of 0.23–0.25), using the McKay method (Vangel 1996), compared to clusters (non-dispersed PEMs), 0.27 with 90% confidence interval of 0.26–0.28) (Table 1). Hence, dispersion helped to develop synchronized embryos in this cell line. However, for cell line 11:12:04 in bioreactors, the coefficients were equal; therefore, dispersion did not improve synchronized maturation of embryos for that cell line.

Cell line 11:12:02 on solid medium showed a CV for the length of mature embryos developed from dispersed PEMs (0.204 with 90% confidence interval of 0.19–0.22) that was smaller than from non-dispersed PEMs (0.271 with 90% confidence interval of 0.25–0.30). Coefficient of variance values for dispersed and non-dispersed aggregates of PEMs were 0.35 and 0.36, respectively, for cell line 09:73:06 (Table 1). Since the distribution of the function φ(q̅) was smaller for dispersed PEMs, it could be concluded that dispersion had a favorable effect on synchronized development of mature embryos. Coefficients of variation for cell lines 11:12:04 and 09:77:03 on solid medium were greater for dispersed PEMs compared to non-dispersed PEMs (Table 1). Based on these CV values, it appeared that dispersion stimulated synchronized development of mature embryos on solid medium for cell line 11:12:02 and 09:73:06, but not for cell lines 11:12:04 and 09:77:03.

Of the harvested mature somatic embryos from dispersed PEMs cultured in bioreactors, 74% (11:12:02) and 50% (11:12:04) germinated and developed roots of 1 cm or longer, and 67% (11:12:02) and 61% (11:12:04) of somatic embryos from non-dispersed PEMs developed to germinants with roots of 1 cm or longer (Table 3). Therefore, more somatic embryos from dispersed PEMs of cell line 11:12:02 germinated, but this was not the case for cell line 11:12:04; 60% (11:12:02) and 68% (11:12:04) of the harvested matured somatic embryos from dispersed PEMs cultured on solid medium germinated and had roots of 1 cm or longer, and 55% (11:12:02) and 67% (11:12:04) of the harvested somatic embryos from non-dispersed aggregates of PEMs grown on solid medium germinated with roots 1 cm or longer (data not shown).

**Table 3.** Germination of mature Norway spruce (*Picea abies*) somatic embryos of cell lines 11:12:02 and 11:12:04, developed from dispersed and non-dispersed pro-embryogenic masses (PEMs) in bioreactors. The number of mature embryos evaluated was pooled from all bioreactors with the same treatment. A sample of mature embryos was collected from each bioreactor, on average 15% in 11:12:02 and 18% in 11:12:04 from bioreactors with dispersed PEMs, and on average 75% in 11:12:02 and 59% in 11:12:04 in bioreactors with non-dispersed PEMs. Germination success was evaluated after 6 wk of germination

Cell line	11:12:02		11:12:04	
	Numbers of mature embryos and germinants			
Culture condition	Dispersed	Non-dispersed	Dispersed	Non-dispersed
Mature somatic embryos collected for germination	1006	994	605	608
Germination started	865 (86%)	922 (93%)	340 (56%)	465 (76%)
Root development ≥ 1 cm after 6 wk	748 (74%)	670 (67%)	303 (50%)	373 (61%)

## Discussion

Aggregation as a factor that reduces overall productivity of a somatic embryo culture in terms of plant yields was first described in carrot (Fujimura and Komamine 1979). Subsequent studies to develop methods for selection of the most productive aggregates and for synchronization of embryo development have been undertaken almost exclusively in carrot (reviewed in Fujimura 2014).

When conifer somatic embryo cultures proliferate, the newly formed somatic embryos cluster together, forming sheets of more or less tightly connected tissue (PEMs). The internal parts of the tissue sheets will eventually be composed of dormant embryos, whereas the actively proliferating PEMs are confined to the surface layers. By breaking up such sheets of PEMs, early-stage somatic embryos and smaller aggregates of embryos are released from the internal parts of the tissue sheets to multiply freely and thereby increase growth rates of the cultures.

In conifer PEM cultures, methods to separate PEM tissue into smaller sizes of aggregates are routinely practiced by agitating PEM tissue in liquid medium, typically in an Erlenmeyer flask, then plating out suspended PEMs onto filter paper for proliferation or maturation (Klimaszewska and Smith 1997; Klimaszewska and Cyr 2002; Lelu-Walter *et al.* 2006; Carneros *et al.* 2009). The results from the present study demonstrated that conifer early-stage somatic embryo (PEMs) cultures could also be dispersed through fluid dynamics strain into smaller units of tissue, to obtain positive effects on maturation yields.

The disperser system used for this study (Mamun 2015) is based on the principal of the disperser system (Aidun and Egertsdotter 2015) used in the automated system for mature embryo harvest (SE Fluidics system; Aidun and Egertsdotter 2012), where fluid dynamics forces are utilized to subject aggregated tissue to axially extensional strain and radially compressional strain to break up the aggregates. During dispersion, the pump was run at a flow rate of 2500 mL min^−1^ under sterile conditions.

Size distribution studies of dispersed Norway spruce PEMs showed that an average of 80% of dispersed PEMs were equal to or less than 0.2 mm^2^ (Fig. 1), indicating that even smaller aggregates could develop into PEMs responsive to maturation treatment. However, it had been previously shown that after sieving of proliferating Norway spruce suspension cultures, only PEMs collected on top of a 0.2-mm mesh (PEM I) were able to develop further into more advanced PEM structures (PEM II and III), whereas the fraction smaller than 0.080 mm did not form any more advanced PEM structures (Filonova *et al.* 2000). Taken together, these data suggested that the fraction of aggregates with an average diameter of between 0.080 and 0.2 mm generated most of the mature embryos in the present study. No information on the size of the aggregates generated by the ‘flask-dispersion’ method discussed above have been found for comparison, although this method was found to produce much better proliferation rates in *Pinus pinea* L. (Stone pine), when compared to pieces of 100 mg tissue (approximately 2-mm-diameter clumps) cultured on solid medium (Carneros *et al.* 2009). In the present study, dispersion and redistribution of immature embryos of Norway spruce had an overall positive influence on maturation in both bioreactors and on solid medium (Fig. 3*a*, *b*). Dispersing methods that break up aggregates by shaking suspension flasks typically involve plating the dispersed culture onto a filter paper after removing the liquid with vacuum, then placing the filter paper onto solidified medium. In the present study, dispersed cultures were transferred onto the mesh of a temporary immersion bioreactor, or directly onto filter paper on the gelled medium surface, still suspended in culture medium, thus differing from the ‘flask-dispersion’ with respect to the vacuum treatment.

In this current study, the variation in length of mature embryos in a culture as an indication of synchronization level was compared. The degree of synchronization was estimated by comparing the differences between values for FWHM examined by the coefficient of variation. The CV, defined as the ratio of standard deviation to the mean, is a useful measure of variation from the mean within a data set (Daniel and Cross 2013). Coefficient of variation of lengths of embryos from dispersed and non-dispersed cultures allowed the testing of the hypothesis of whether lengths of embryos from non-dispersed PEMs were more widely distributed, compared to lengths of embryos from dispersed PEMs. The 90% confidence interval for the CVs was computed by the McKay method (Vangel 1996). The average embryo lengths were found to be significantly more focused around the mean in dispersed than non-dispersed PEMs only in cell line 11:12:02 cultured in bioreactors (Table 1). Thus, it appeared that synchronization in lengths of mature embryos present in a culture was not always linked to embryo yield, as all four cell lines showed significantly increased mature embryo yields after dispersion, but not the corresponding increase in uniformity in length of embryos, compared to the non-dispersed cultures. However, the cell line 11:12:02 in bioreactors that did show increased synchronization by less widely distributed embryo lengths overall showed the best performance in response to dispersion with the highest increases in embryo yield, length of embryos, and germination rates, indicating that synchronization of mature embryos is positively linked to embryo yields.

Liquid suspension culture of conifer PEMs offers advantages over solid culture medium by producing higher rates of multiplication and less aggregation (von Arnold *et al.* 2002). The benefits from liquid culture medium for scale-up production of conifer PEMs have also been acknowledged (Gupta and Timmis 2005). Successful conversion of PEMs to mature embryos fully submerged in liquid medium in a suspension culture has been reported for *Picea glauca* (Moench) Voss (white spruce; Hakman and von Arnold 1988) and for Norway spruce (Gorbatenko and Hakman 2001). However, in general, it is not possible to get high maturation yields from fully submerged conifer PEM cultures. Early-stage somatic embryos of Norway spruce in rotated suspension culture do not form large polarized embryos, with an embryonic region and suspensor cells protruding unidirectionally, but instead the larger embryo structures manifest in a spherical radiating shape (Egertsdotter and von Arnold 1993). It appears that PEMs need a solid support for forming the later-stage polarized PEM structures capable of responding to maturation treatment. To further elucidate the potential negative effects of liquid culture conditions on somatic embryo development, the effects of fluid shear stress on development of PEMs have been studied in a flow cell system. It was demonstrated that maintaining PEMs under shear stress from fluid flow conditions prevents directional growth of the PEMs (Sun *et al.* 2010) and furthermore, that PEMs under such conditions are less likely to mature (Sun *et al.* 2011). Temporary immersion bioreactor systems offer an ideal liquid-based system, providing benefits from liquid culture medium with respect to accessibility to culture components. These bioreactors also provide a support surface for proper growth and development of embryos, and the possibilities to supply air or gases as needed to avoid vitrification (Mamun *et al.* 2014). Here, increased embryo yields from one of two cell lines tested in bioreactors relative cultures on solid medium was demonstrated for Norway spruce. The prospects of using bioreactors for large-scale production of mature embryos offer advantages over solid medium cultures in terms of reduced labor, less waste of plastic consumables, and the possibilities for scale-up by automation.

## Conclusion

In the present study, the positive effect of dispersion of proliferating, immature, somatic embryo-aggregates on the yield of mature embryos was demonstrated in Norway spruce cultures. On average across cell lines, the yields of mature embryos per fresh weight PEMs after dispersion were more than three times (in liquid medium) and two times (in solid medium) higher than that of the non-dispersed controls. This was true except for cell line 11:12:04, which did not show higher yields of mature embryos after dispersion, when cultured on solid medium. Furthermore, in bioreactors, dispersion significantly improved length and synchronization of mature embryos in cell line 11:12:02, but not cell line 11:12:04. On solid medium, none of the tested cell lines showed significantly increased embryo lengths in response to dispersion, but two lines showed an increased degree of synchronization. The correlation between synchronization, as measured by distribution of embryo lengths in the culture, and yields of mature embryos, was therefore not clear, as only cell line 11:12:02 showed increased synchronization after dispersion in bioreactors. The present study still demonstrated the benefits of dispersion, compared to non-dispersion, as a tool to increase mature embryo yields. Future studies will focus on other morphological details in addition to embryo length, to better understand the correlation between embryo morphology and the increase in mature embryo yield as an effect of dispersion of the PEMs prior to maturation treatment in conifer cultures.

## Electronic supplementary material

ESM 1 (DOCX 1.95 MB)
