# Supplementary material for: Improved and synchronized maturation of Norway spruce (Picea abies (L.) H.Karst.) somatic embryos in temporary immersion bioreactors
Source: In Vitro Cell Dev Biol Plant. 2018 Jul 20;54(6):612–20. doi: 10.1007/s11627-018-9911-4 (PMC6223745; doi:10.1007/s11627-018-9911-4)

**Supplemental materials.**

**Figure 1s.**Method for evaluation of degree of synchronization through embryo length measurement

Nomenclature

$D\left( \bar{q} \right)$ Sum of the lengths of all embryos in a length interval, $\delta$

$n$ Number of mature embryos in a length interval, $\delta$

$q_{i}$ Length of an embryo *i*

$\bar{q}$ Average length of somatic embryos in a length interval, $\delta$

$\varphi\left( \bar{q} \right)$ Normalized distribution function

Description of length measurements

The developmental stage of an embryo in terms of level of development parameter,$q_{i}$, where in this study it was assumed $q_{i}$ represented the length of an embryo. To evaluate the level of synchronization of embryo growth and development, it was assumed that the average length of somatic embryos with a length interval, $\delta$, was defined as

$\bar{q}\equiv\frac{\sum_{i=1}^{n} q_{i}}{n}$ , then

$Q\equiv\int_{0}^{\infty} D\left( \bar{q} \right)d\bar{q}$.

The normalized distribution function was defined as$\varphi\left( \bar{q} \right)\equiv\frac{D(\bar{q})}{Q}$, then

$\int_{0}^{\infty} \varphi\left( \bar{q} \right)d\bar{q}=1$.

The full width at half maximum (FWHM) (*i.e.* the full width of $\varphi\left( \bar{q} \right)$ at 50% of maximum), 66%, and 75% of maximum of the function $\varphi\left( \bar{q} \right)$ as a measure of synchronization.

**Figure 2s** Level of synchronized development of somatic embryos of Norway spruce (*Picea abies*) in liquid medium in bioreactors. These plots are for cell lines 11:12:02 (*a – l*), and 11:12:04 (*m – v*) cultured in different bioreactors. Plots (*a – f* and *m – q*) represent the distribution function, $\varphi\left( \overline{q} \right)$, with respect to the average length in 1 mm interval of mature somatic embryos formed from dispersed PEMs. Similarly plots (*g – l* and *r – v*) represent that of in controls. I, II, and III represent full width at half (50%), 66%, and 75% of maximum of the function $\varphi\left( \overline{q} \right)$ respectively. Their pertaining values are (a) 1.5, 1.15, & 0.92 mm; (b) 1.25, 0.95, & 0.78 mm; (c) 1.65, 1.23, & 1.0 mm; and (d) 1.4, 1.0, & 0.83 mm; (e) 1.42, 1.0, 0.82 mm; (f) 1.6, 1.17, & 0.95 mm; (g) 1.5, 1.15, & 0.92 mm; (h) 1.85, 1.55, & 1.33 mm; (i) 1.68, 1.24, & 0.97 mm; (j) 1.93, 1.43, & 1.17 mm; (k) 1.45, 1.08, & 0.88 mm; (l) 1.5, 1.1, & 0.88 mm; (m) 1.88, 1.65, & 1.35 mm; (n) 1.9, 1.5, & 1.2 mm; (o) 1.8, 1.3, & 1.05 mm; (p) 2.5, 1.85, & 1.55 mm; (q) 1.9, 1.4, & 1.15 mm; (r) 1.65, 1.25, & 1.0 mm; (s) 1.87, 1.5, & 1.25 mm; (t) 2.2, 1.85, & 1.64 mm; (u) 1.88, 1.5, &1.2 mm; (v) 1.45, 1.07, & 0.86 mm.


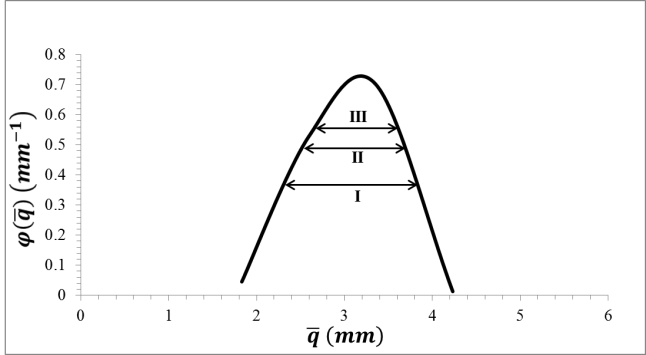


(a)


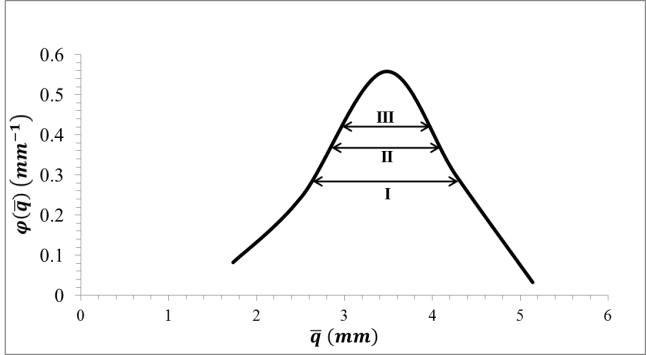


(c)


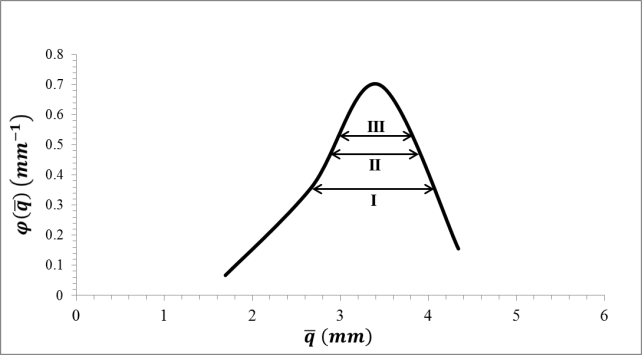


(d)


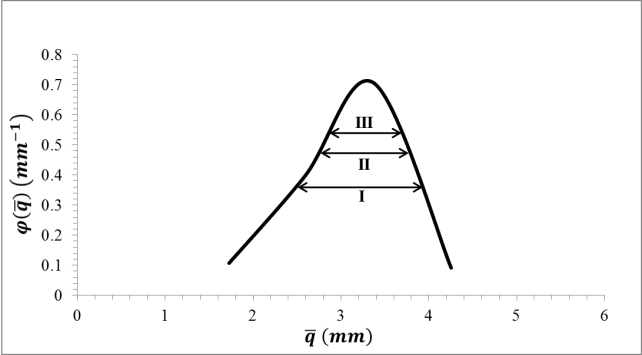


(e)


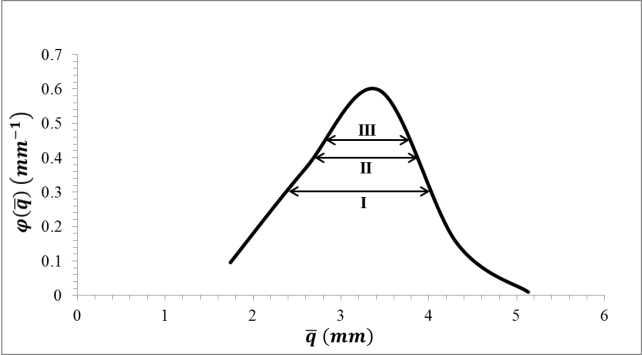


(f)


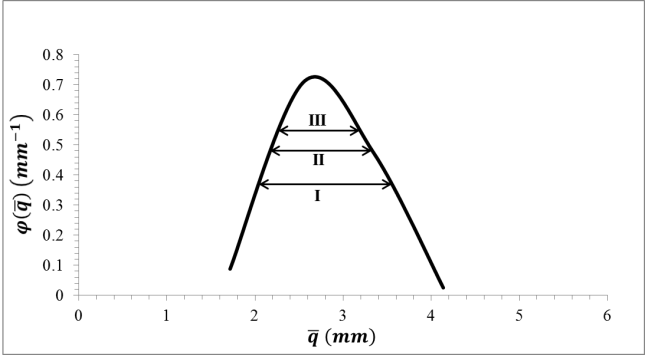


(g)


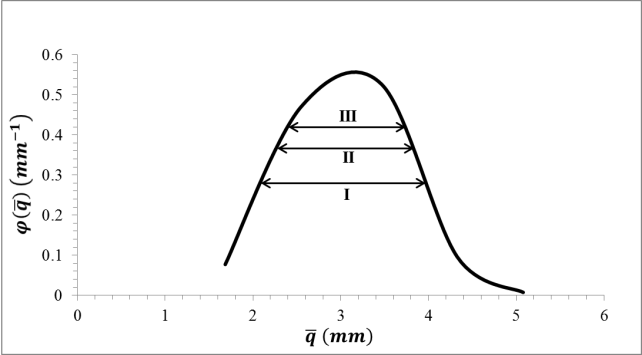


(h)


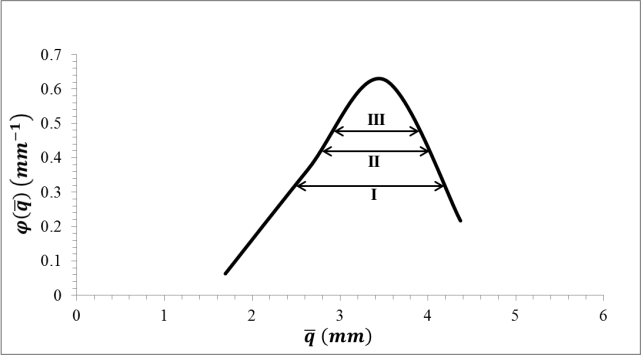


(i)


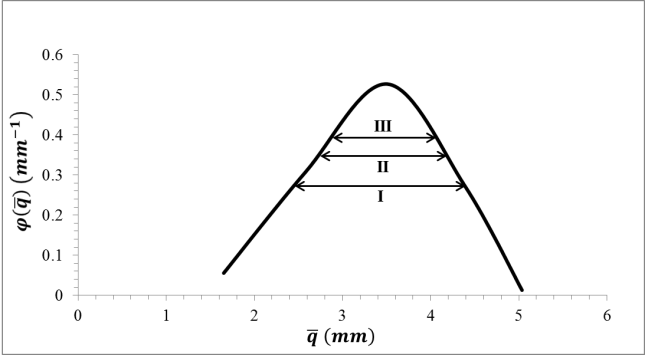


(j)

(b)


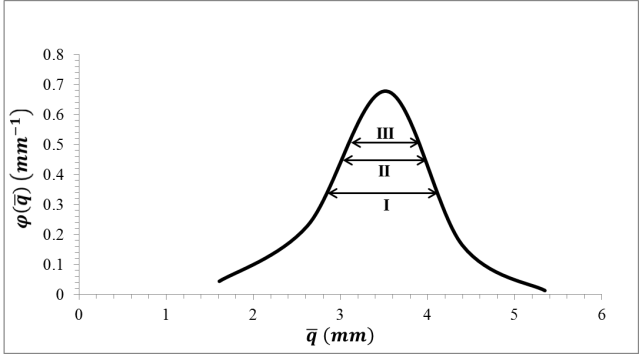


**Figure 2s contontinued.**


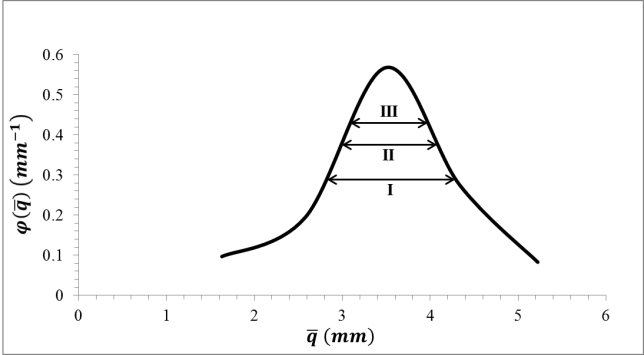


(k)


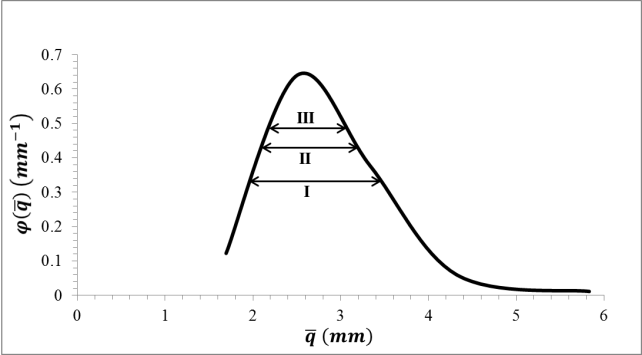


(l)


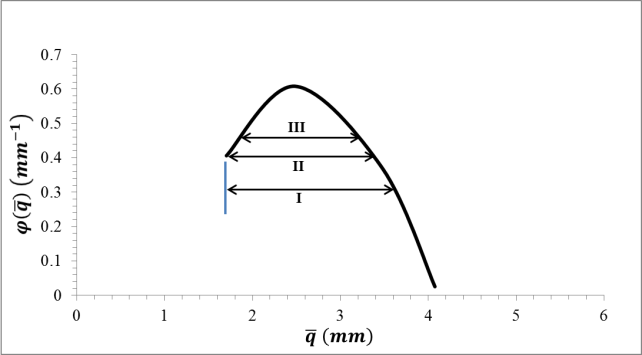


(m)


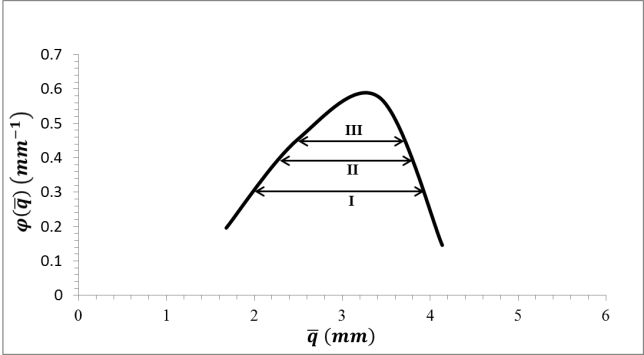


(n)

(o)


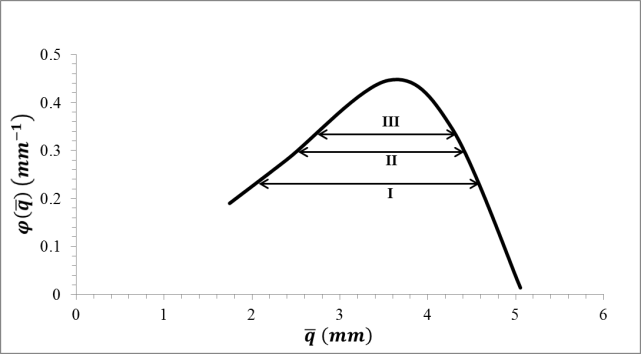


(p)


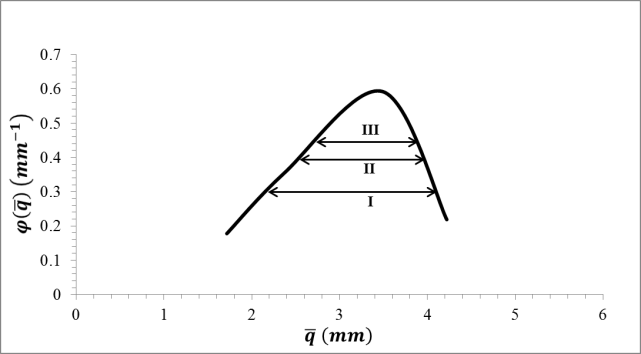


(q)


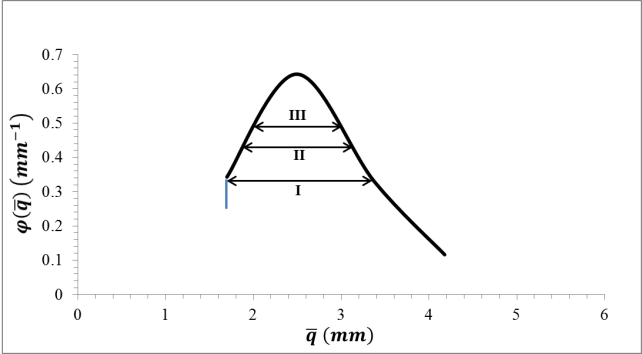


(r)


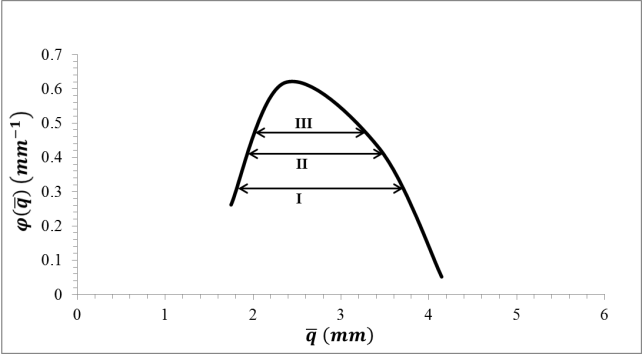


(s)


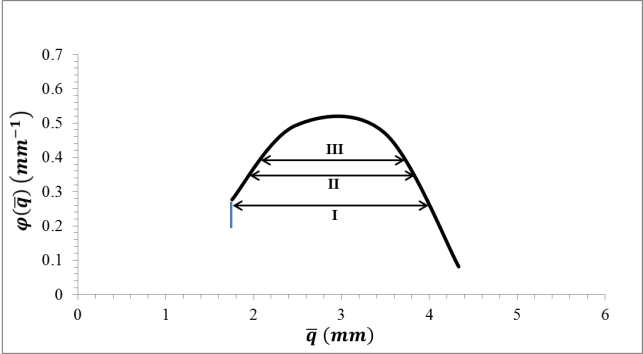


(t)


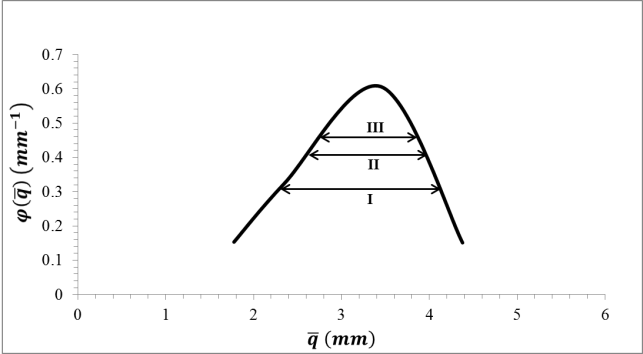


**APPENDIX 3**


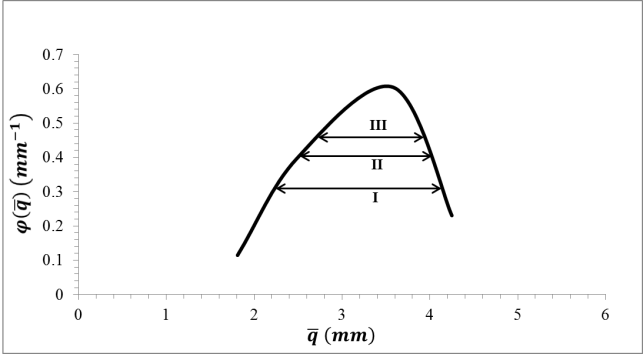


(u)


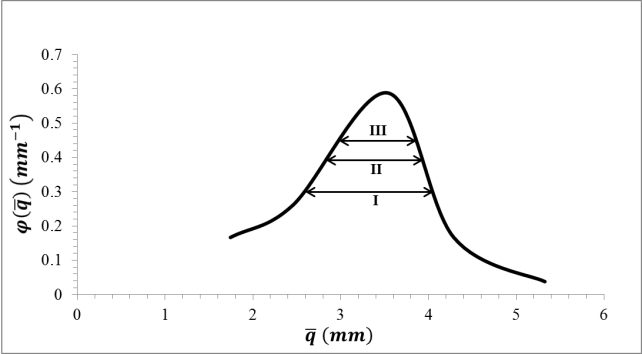


(v)

Figure 3s. Level of synchronized development of mature somatic embryos of Norway spruce (*Picea abies*) of cell lines 11:12:02 (*a, b*), 11:12:04 (*c, d*), 09:73:06 (*e, f*), and 09:77:03 (*g, h*) in solid medium. Plots (*a, c, e,* and *g*) represent the distribution function, $\varphi\left( \overline{q} \right)$, with respect to the average length in an interval, $\delta$, of mature somatic embryos formed from dispersed PEMs in a replicate of the corresponding cell line. Similarly plots (*b, d, f,* and *h*) represent that of in controls. I, II, and III represent full width at half (50%), 66%, and 75% of maximum of the function $\varphi\left( \overline{q} \right)$ respectively. Their pertaining values are (a) 0.8, 0.58, & 0.48 mm; (b) 1.7, 1.4, & 1.2 mm; (c) 1.5, 1.22, & 1.0 mm; (d) 1.03, 0.75, & 0.58 mm; (e) 2.0, 1.42, & 1.05 mm; (f) 2.6, 1.95, & 1.58 mm; (g) 1.45, 1.15, & 0.93 mm; and (h) 1.18, 0.88, & 0.7 mm.


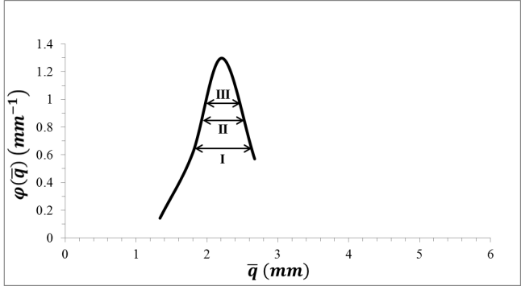


(a)


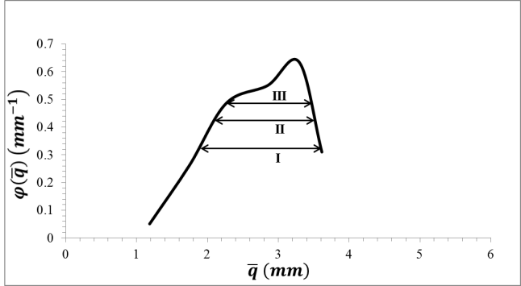


(b)


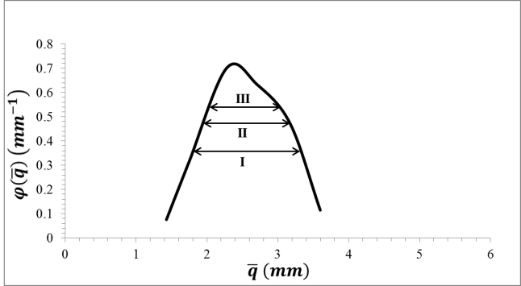


(c)


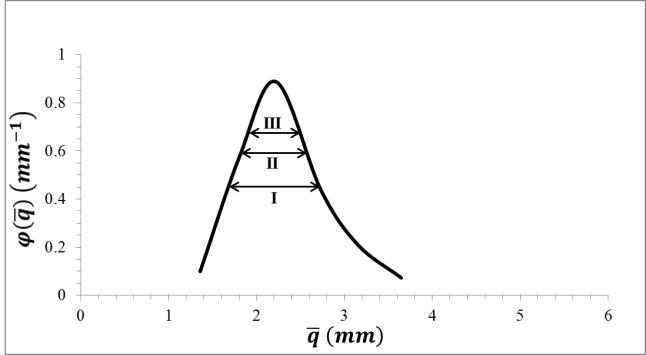


(d)


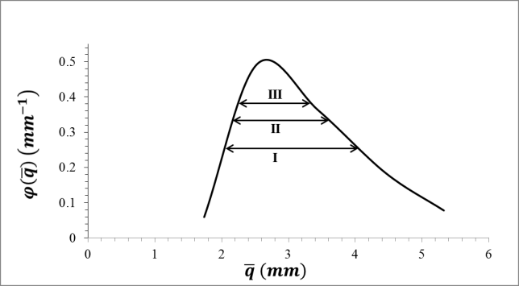


(e)


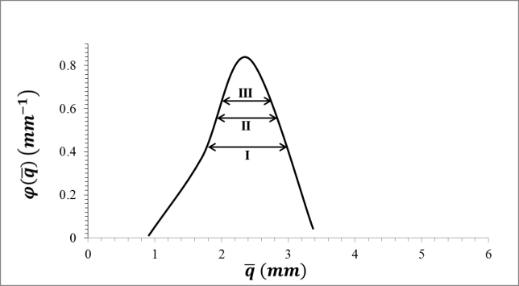


(h)

(g)


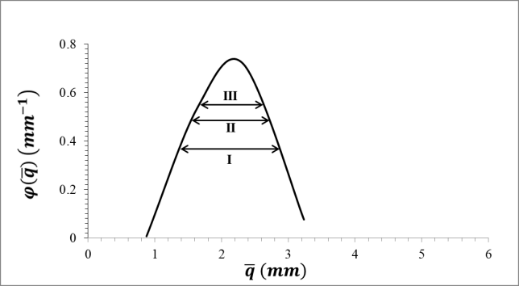


(f)


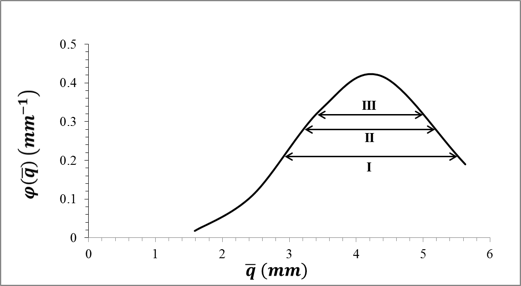

Supplement: Supplementary file 1 — (DOCX 1.95 MB) [file 11627_2018_9911_MOESM1_ESM.docx]
